# Acute pain management in children: a survey of Italian pediatricians

**DOI:** 10.1186/s13052-019-0754-3

**Published:** 2019-12-03

**Authors:** Gian Luigi Marseglia, Maria Alessio, Liviana Da Dalt, Maria Giuliano, Angelo Ravelli, Paola Marchisio

**Affiliations:** 1 Pediatrics Clinic, Pediatrics Department, Policlinico San Matteo, University of Pavia, viale Golgi 19, Pavia, Italy; 2 Pediatrics Clinic, Pediatrics Department, Federico II University, Via Sergio Pansini 5, Naples, Italy; 3 Department of Woman and Child Health, University of Padua, Via Giustiniani 2, Padua, Italy; 4 Azienda Sanitaria Locale Napoli 3, Naples, Italy; 5 Università degli Studi di Genova and IRCCS Istituto Giannina Gaslini, via Gerolamo Gaslini 5, Genoa, Italy; 6 Fondazione IRCCS Ca’ Granda Ospedale Maggiore Policlinico and Department of Pathophysiology and Transplantation, University of Milan, via Francesco Sforza, 28, Milan, Italy

**Keywords:** Acetaminophen, Algometric scale, Ibuprofen, Italy, Pain, Pediatrics, Survey

## Abstract

**Background:**

Current guidelines recommend assessing and relieving pain in all children and in all instances; yet, in clinical practice, management is frequently suboptimal. We investigated the attitude of Italian family pediatricians towards the evaluation and treatment of different types of acute pain in children aged 7–12 years.

**Methods:**

This is a cross-sectional study based on a 17-question survey accessible online from October 2017 to October 2018. Responders had to describe cases of children suffering from any type of acute pain among headache, sore throat, musculoskeletal/post-traumatic pain, and earache. Children’s characteristics, pain assessment modalities and therapeutic approaches were queried. The following tests were used: Z-proportion to evaluate the distribution of categorical data; chi-squared and Kruskall-Wallis to explore data heterogeneity across groups; Mann-Whitney for head-to-head comparisons.

**Results:**

Overall, 929 pediatricians presented 6335 cases uniformly distributed across the types examined. Pain was more frequently of moderate intensity (42.2%, *P* < 0.001) and short duration (within some days: 98.4%, *P* < 0.001). Only 50.1% of responders used an algometric scale to measure pain and 60.5% always prescribed a treatment. In children with mild-moderate pain (*N* = 4438), the most commonly used first-line non-opioids were ibuprofen (53.3%) and acetaminophen (44.4%). Importantly, a non-recommended dosage was prescribed in only 5.3% of acetaminophen-treated cases (overdosing). Among the misconceptions emerged, there were the following: i) ibuprofen and acetaminophen have different efficacy and safety profiles (when choosing the non-opioid, effectiveness weighted more for ibuprofen [79.7% vs 74.3%, *P* < 0.001] and tolerability for acetaminophen [74.0% vs 55.4%, *P* < 0.001]); ii) ibuprofen must be taken after meals to prevent gastric toxicities (52.5%); ibuprofen and acetaminophen can be used combined/alternated for persisting mild-moderate pain (16.1%). In case of moderate-severe pain not completely controlled by opioids, ibuprofen and acetaminophen were the most used add-on medications, with ibuprofen being much more prescribed than acetaminophen (65.2% vs 23.7%, respectively) overall and in all pain types.

**Conclusions:**

Several gaps exist between the current practice of pain assessment and treatment and recommendations. Further efforts are needed to raise awareness and improve education on the possible exposure of the child to short- and long-term consequences in case of suboptimal pain management.

## Background

Pain is a very common symptom in children of all ages. It is a multidimensional phenomenon characterized by sensory, physiological, affective, cognitive, behavioral and spiritual aspects and it is defined by the International Association for the study of Pain as “an unpleasant sensory and emotional experience associated with actual or potential tissue damage, or described in terms of such damage” [1]. Independently of the cause and patient age, pain may weaken the physical and psychological integrity of the child and cause stress to the parents; moreover, if left untreated, it may exert short- and long-term effects, including sensitization to pain episodes later in life [2, 3]: for all these reasons, pain should be assessed and treated in every patient [4–10].

Despite the availability of national and international guidelines, in clinical practice pain remains often under-recognized, under-evaluated and under-treated [11–19]. In Italy, the law 38/2010, concerning the right to access palliative and pain care for all patients, has focused the attention of pediatricians on the importance of considering the specific needs of children and their families during the course of the disease. Consequently, many initiatives have been undertaken to evaluate the pragmatic approach to pain management in children, including a number of meetings, courses, and surveys promoted by the Federation of Italian Pediatricians. In particular, in June 2011, a survey based on an anonymous questionnaire aimed to evaluate the modalities of pain assessment was sent online to 4900 family pediatricians, and re-administered in February 2013 following a 3-day training-qualification course [20]. The results showed increased awareness of insufficient pain assessment in children after the course (from 47 to 53%), relevant improvement in the practice of measuring and reporting pain in medical records (from 58 to 74%), and marked increase in the use of age-related algometric scales (from 10 to 43%) [20]. However, although the change in the attitude was remarkable, it was not sufficient to ensure the optimal management of pain in the pediatric population.

To provide an update on Italian family pediatricians’ attitude towards the assessment and treatment of acute pain in school-aged children, a new survey was undertaken in 2018. The aim of this study was to understand the knowledge/misconceptions regarding the management of different types of acute pain and to assess the adherence of current practice to the available recommendations and clinical evidence.

## Methods

This is an Italian cross-sectional study based on a survey conducted as part of a continuing medical education (CME) course entitled “Acute Pain Management in Pediatrics”. This distance learning course organized by Edra (Milan, Italy), was accessible online between 25 October 2017 and 24 October 2018 and involved family pediatricians. The CME course was promoted via a voucher, in accordance with the CME regulation. Participation in the CME course was voluntary and completion of the course and a positive result in the final test allowed participants to obtain training credits.

The questionnaire, formulated by the authors of the CME course (included among the authors of this article), consisted of a total of 17 questions. All the answers were mandatory and participants who wanted to complete the CME course had to fill in at least two questionnaires for each type of pain (headache, sore throat, musculoskeletal and post-traumatic pain, and earache).

All questionnaires were completely anonymous and, apart from the detailed information below, no personally identifiable information was collected. The questionnaire path varied according to the selected answers (see details in the translated questionnaire in Additional file 1).

The survey included as first step the selection of the type of pain, followed by 17 questions: to answer, the pediatrician was asked to refer to a clinical scenario of a child with acute pain managed in his/her daily practice. Only children aged 7–12 years were considered. Questions 1–4 analyzed the characteristics of the pain (i.e. intensity and duration) and the child demographics of the cases described, questions 5–6 collected information on pain assessment, and the remaining questions addressed the therapeutic approach adopted in general (question 7), and for the management of the case presented (questions 8–17).

Data obtained from the answers were reported as number and percentage (N, %) for categorical variables. The answers expressed by scores were reported as mean, median, standard deviation (SD) and interquartile range (IQR). The Z-proportion test was used to evaluate the distribution of categorical data. The chi-squared test and Kruskall-Wallis test were used to evaluate the heterogeneity of data across groups. The Mann-Whitney test was used for head-to-head comparisons. The *P*-value was adjusted by means of the false discovery rate method for multiple comparisons; *P*-values < 0.05 were considered significant. All analyses were carried out through software R version 3.5.2.

## Results

### Study participants

Overall, 929 pediatricians completed at least 1 questionnaire for a total of 6335 cases, with 777 physicians including at least 8 cases. The main characteristics of study participants are listed in Table 1.

**Table 1:** Main characteristics of participants

Total number	929
Gender	
Males	295 (32)
Females	634 (68)
Age group, years	
31–40	73 (7.9)
41–50	116 (12.5)
51–60	322 (34.6)
61–70	413 (44.5)
> 70	5 (0.5)
Geographical area^a^	
Northwest	211 (34.9)
Northeast	40 (6.6)
Centre	184 (30.5)
South	169 (28)
Questionnaires completed, median (range)	8 (1–17)

Data are expressed as frequencies (*N* [%]), unless otherwise specified

^a^Geographical area was not a mandatory field. % is calculated on 604 answers received

A similar number of cases for each pain type was described by responders: 1596 (25.2%) with headache, 1593 (25.2%) with sore throat, 1546 (24.4%) with musculoskeletal or post-traumatic pain, and 1600 (25.2%) with earache.

### Survey results

#### Q1-Q4: characteristics of acute pain clinical scenarios described in the questionnaires

Data on pain severity and duration and by pain type (*question 1 and 2*) are shown in Table 2: pain was significantly more frequently of moderate intensity (*N* = 2673, 42.2%, *P* < 0.001) and short duration (within some days in 6231 [98.4%] cases, *P* < 0.001). Both intensity and duration were significantly correlated with pain type (*P* < 0.001, although for the duration of headache and musculoskeletal pain the trend was not as clear as for the other pain types).

**Table 2:** Characteristics of acute pain clinical scenarios described in the questionnaires

Characteristic	Type of pain					Total
	Headache *N* = 1596	Sore throat *N* = 1593	Musculoskeletal or post-traumatic *N* = 1546	Earache *N* = 1600	*P*-value	*N* = 6335
Intensity, *N* (%)						
Mild	572 (35.8)	485 (30.5)	384 (24.8)	324 (20.3)	< 0.001	1765 (27.9)
Moderate	674 (42.2)	701 (44.0)	679 (43.9)	619 (38.7)		2673 (42.2)
Severe	350 (22.0)	407 (25.5)	483 (31.3)	657 (41.9)		1897 (29.9)
Duration, *N* (%)						
A few hours	700 (43.9)	344 (21.6)	458 (29.6)	875 (54.7)	< 0.001	2377 (37.5)
1 day	373 (23.4)	593 (37.2)	414 (26.8)	472 (29.5)		1852 (29.2)
Some days	482 (30.2)	645 (40.5)	625 (40.4)	250 (15.6)		2002 (31.7)
At least 1 week	41 (2.5)	11 (0.7)	49 (3.2)	3 (0.2)		104 (1.6)

Data are expressed as frequencies (*N* [%])

Of the 6635 children with acute pain described by responders (*question 3–4*), 3487 (55%) were females, and 1383 (21.8%) were aged 7, 1444 (22.8%) 8, 938 (14.8%) 9, 1235 (19.5%) 10, 607 (9.6%) 11 and 728 (11.5) 12 year.

#### Q5-Q6: pain measurement

**Fig. 1:**
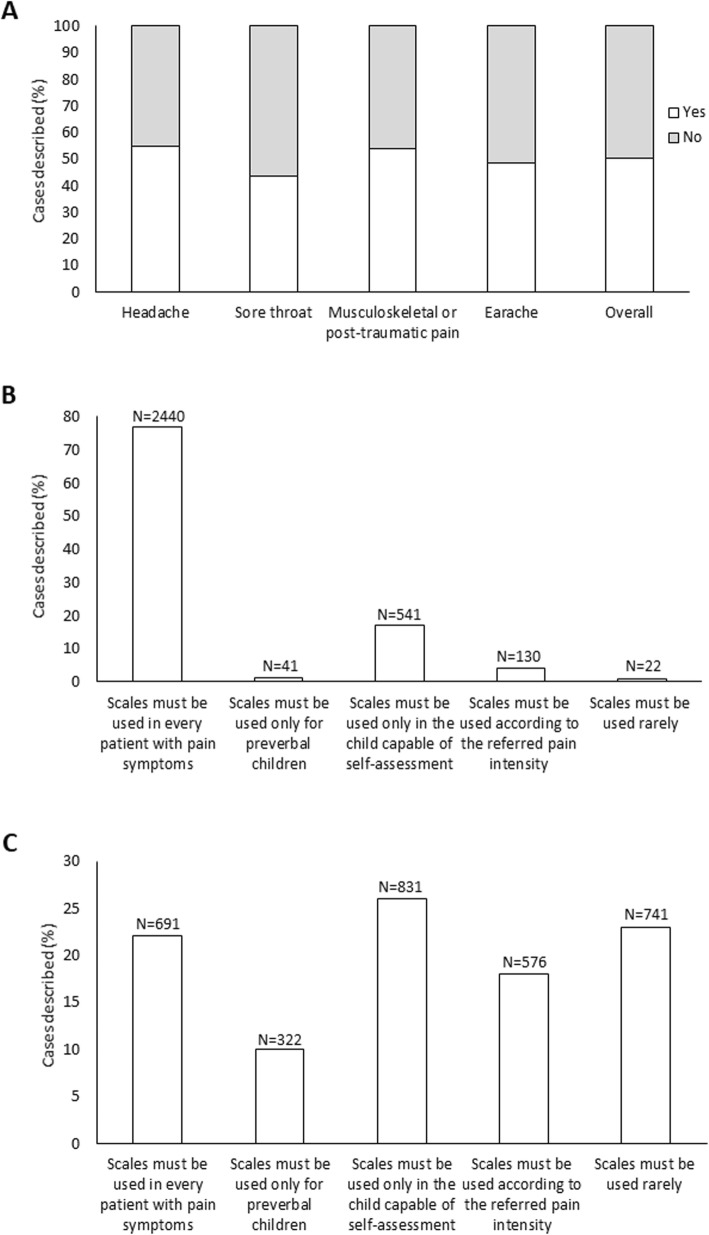
**a** Cases in which an algometric scale was/was not used to measure pain, as a whole and by pain type. **b** Factors driving the choice of using an algometric scale. **c** Factors driving the choice not to use an algometric scale

An algometric scale for pain measurement was used in 3174/6335 (50.1%) cases (Fig. 1a), and in most of them (2440/3174, 76.9%) the pediatricians made this choice because they felt that such tool must be used in every patient with pain symptoms (Fig. 1b). In contrast, the main reason for not employing an algometric scale was that it should be applied only in children able to self-assess pain (831/3161, 26.3%) or just occasionally (741/3161, 23.4%) (Fig. 1c). An algometric scale was used more frequently for measuring headache (54.8%, *P* < 0.001) and musculoskeletal pain or traumatic pain (53.9%, *P* = 0.002), and less frequently for sore throat (43.6%, *P* < 0.0001).

#### Q7-Q17: therapeutic approach

Next, participants were asked to indicate the main factors that, in general, drive their decision to treat pediatric pain (*question 7*): 3831/6335 (60.5%) stated that they always prescribe a treatment, as pain is a negative experience for the child, whereas 1976/6335 (31.2%) declared to prescribe an analgesic only for severe pain. Answers were similar by pain type.

The criteria rated as important/very important in the decision to start an analgesic therapy (*question 8*) were the impact of pain on the quality of life in 4990/6335 cases (78.8%) and pain intensity in 4845/6335 (76.5%), followed by pain origin in 3851/6335 (60.8%) and duration in 3638/6335 (57.4%). The scores obtained were compared by the Mann-Whitney test, which indicated a statistically significant difference in terms of importance for the impact on quality of life and pain intensity compared to pain origin and duration; however, while the difference was significant because of the big sample size, the clinical relevance was low, due to the small magnitude of the difference among mean and median score (data not shown).

#### Mild-moderate pain

In children with mild-moderate pain (*question 9*), the general health status was deemed an important/very important driver in the choice of the first-line non-opioid molecule in 3067/4438 (69.1%) cases, followed by age, possible comorbidities and concomitant therapies in 2346 (52.9%), 2288 (51.6%) and 2315 (52.1%), respectively.

**Fig. 2:**
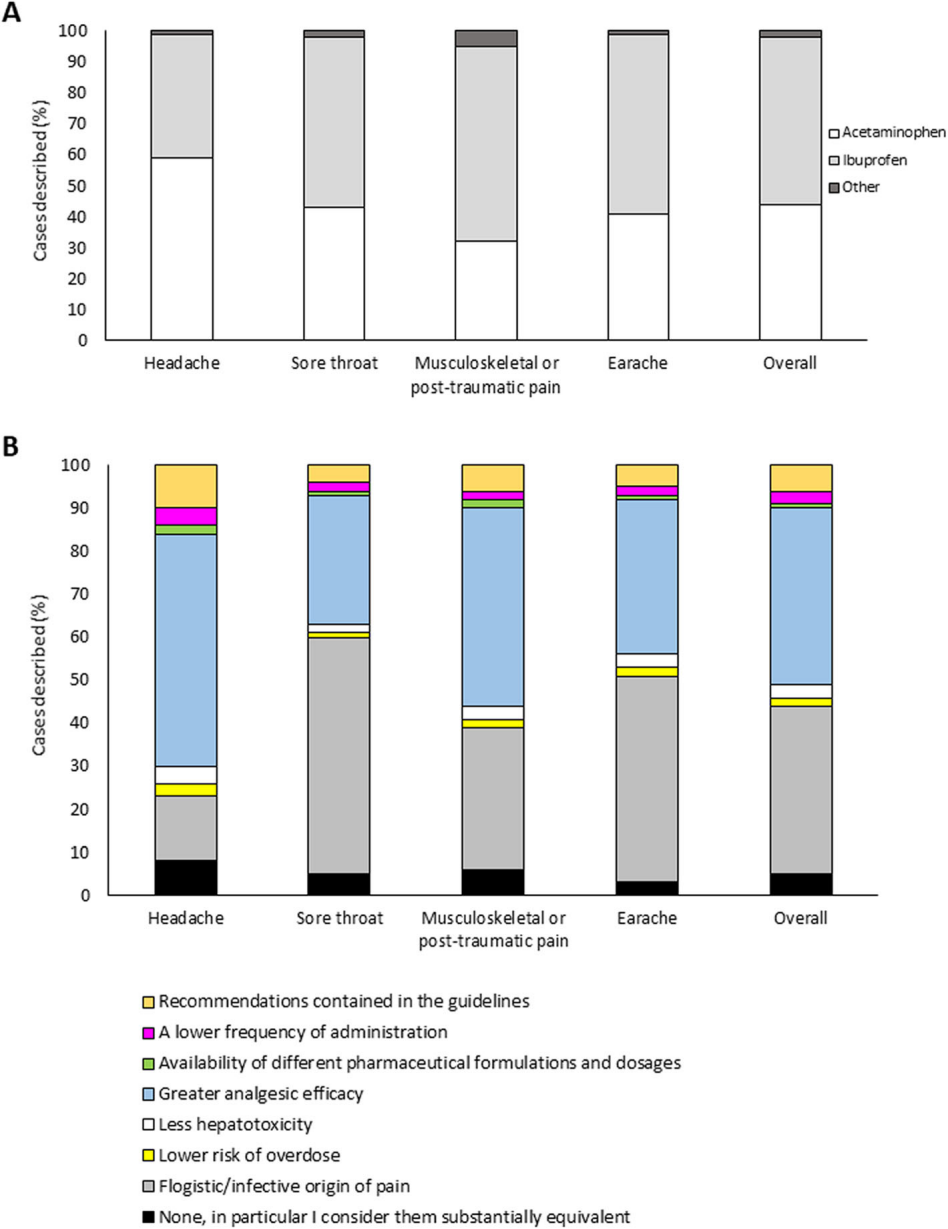
**a** Most frequently prescribed active ingredients, as a whole and by pain type. **b** Reasons driving prescription towards ibuprofen rather than acetaminophen or another painkiller

Overall, the active ingredients most frequently prescribed as oral antalgic non-opioid therapy (*question 10*) were ibuprofen in 2364/4438 (53.3%) cases and acetaminophen in 1972/4438 (44.4%) (Fig. 2a). Ketoprofen lysine salt, naproxen and others were among the possible answers: as they were used in 62 (1.4%) and 14 (0.3%) and 26 (0.6%) of the cases described, respectively, data on these agents were excluded. Ibuprofen was the most frequently administered medication orally in case of musculoskeletal/post-traumatic pain (64%), earache (58%) and sore throat (54%), whereas acetaminophen was most commonly chosen for (treating) headache (59%).

With regard to the dose regimen prescribed (*question 11*, Table 3), it was correct in all children treated with ibuprofen and in 94.7% of those treated with acetaminophen; in the remaining cases (104/1972, 5.3%), the child received a higher-than-recommended dose of acetaminophen (≥15 mg/kg every 4–6 h).

**Table 3:** Dose regimens of ibuprofen and acetaminophen

Medication	Posology	*N* (%)
Ibuprofen, *N* = 2364	5 mg/kg every 6–8 h	912 (36.8)
	10 mg/kg every 6–8 h	1452 (61.4)
Acetaminophen, *N* = 1972	10 mg/kg every 4–6 h	691 (35.0)
	15 mg/kg every 4–6 h	1177 (59.7)
	≥15 mg/kg every 4–6 h	104 (5.3)

Data are expressed as frequencies (*N* [%])

Participants were then asked to rate by importance the reasons for choosing acetaminophen vs ibuprofen as preferred medication (*question 10*). For each reason, the degree of importance (low [score 1–3] vs high [score 4–5]) was similar between acetaminophen and ibuprofen: efficacy, better tolerability, recommendations and common practice were assigned high importance, whereas parental satisfaction/experience and history of asthma were considered of low importance. For acetaminophen, efficacy profile and good tolerability were equally rated as the most important factors (1465/1972 [74.3%] and 1458/1972 [74.0%], respectively), followed by guideline recommendations (*N* = 1231, 62.4%) and common practice (*N* = 1173, 59.5%). For ibuprofen, efficacy was the most important factor in the vast majority of cases (1885/2364 [79.7%]), followed by other recommendations, better tolerability and common practice (*N* = 1354 [57.3%], *N* = 1309 [55.4%] and *N* = 1251 [53.0%], respectively). Parental satisfaction/experience was deemed less important in 924 (46.9%) cases for acetaminophen and in 967 (40.9%) cases for ibuprofen; similar figures were seen for history of asthma (566 [28.7%]) and 527 (22.3%) of cases, respectively).

As reported in Table 4, except for the efficacy profile, that was significantly more relevant in the choice of ibuprofen over acetaminophen, all the other reasons were significantly more relevant in the choice of acetaminophen (chi-squared *P*-value ≤0.001): in particular, better tolerability had a higher weight for 74.0% of pediatricians who prescribed acetaminophen vs 55.4% of those choosing ibuprofen.

**Table 4:** Criteria driving the choice toward ibuprofen or acetaminophen in case of mild-moderate pain

	Acetaminophen *N* = 1972	Ibuprofen *N* = 2364	Chi-squared *P*-value
Effectiveness	1465 (74.3)	**1885 (79.7)**	< 0.001
Better tolerability	**1458 (74.0)**	1309 (55.4)	< 0.001
Recommendations contained in the guidelines	**1231 (62.4)**	1354 (57.3)	0.001
Established therapeutic practice	**1173 (59.5)**	1251 (53.0)	< 0.001
Parental satisfaction / experience	**924 (46.9)**	967 (40.9)	< 0.001
History of asthma	**566 (28.7)**	527 (22.3)	< 0.001

Only answers rated of high importance (i.e. score 4 and 5) are presented. For each drug, the reason(s) with a significantly higher weight in the choice (chi-squared *P*-value) are highlighted in bold.

Data are expressed as frequencies (*N* [%]).

In general, among the possible reasons to prefer ibuprofen over acetaminophen or another painkiller (*question 13*), the significantly more common were its higher efficacy (961/2364, 40.7% *P* < 0.001) and the inflammatory/infective origin of pain (925/2364, 39.1% *P* < 0.001) (Fig. 2b).

In 1241 (52.5%) out of the 2364 clinical scenarios treated with oral ibuprofen, pediatricians stated that they advised to take this medication after meals (*question 14*) because of the fear of gastric side effects; this was also for 332 (14.0%) cases with persistent pain requiring repeated administrations, for 184 (7.8%) cases on long-term therapy and for 109 (4.6%) cases with previous history of gastrointestinal discomfort. In contrast, the prescription not to take ibuprofen after feeding was made when the drug was administered only if needed in 498 (21.1%).

**Fig. 3:**
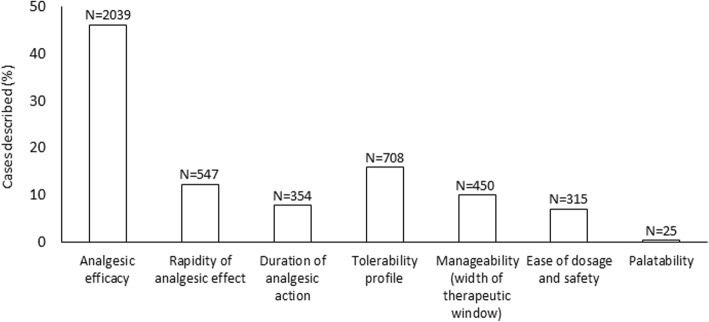
Pharmacologic feature deemed as the most important in the choice of the non-opioid analgesic

Overall, in the cases described in the questionnaires, analgesic efficacy was the pharmacologic feature deemed most important in the choice of the molecule prescribed (in 2039/4438 cases, 45.9%) (Fig. 3), with no significant differences by pain type (*question 15*).

**Fig. 4:**
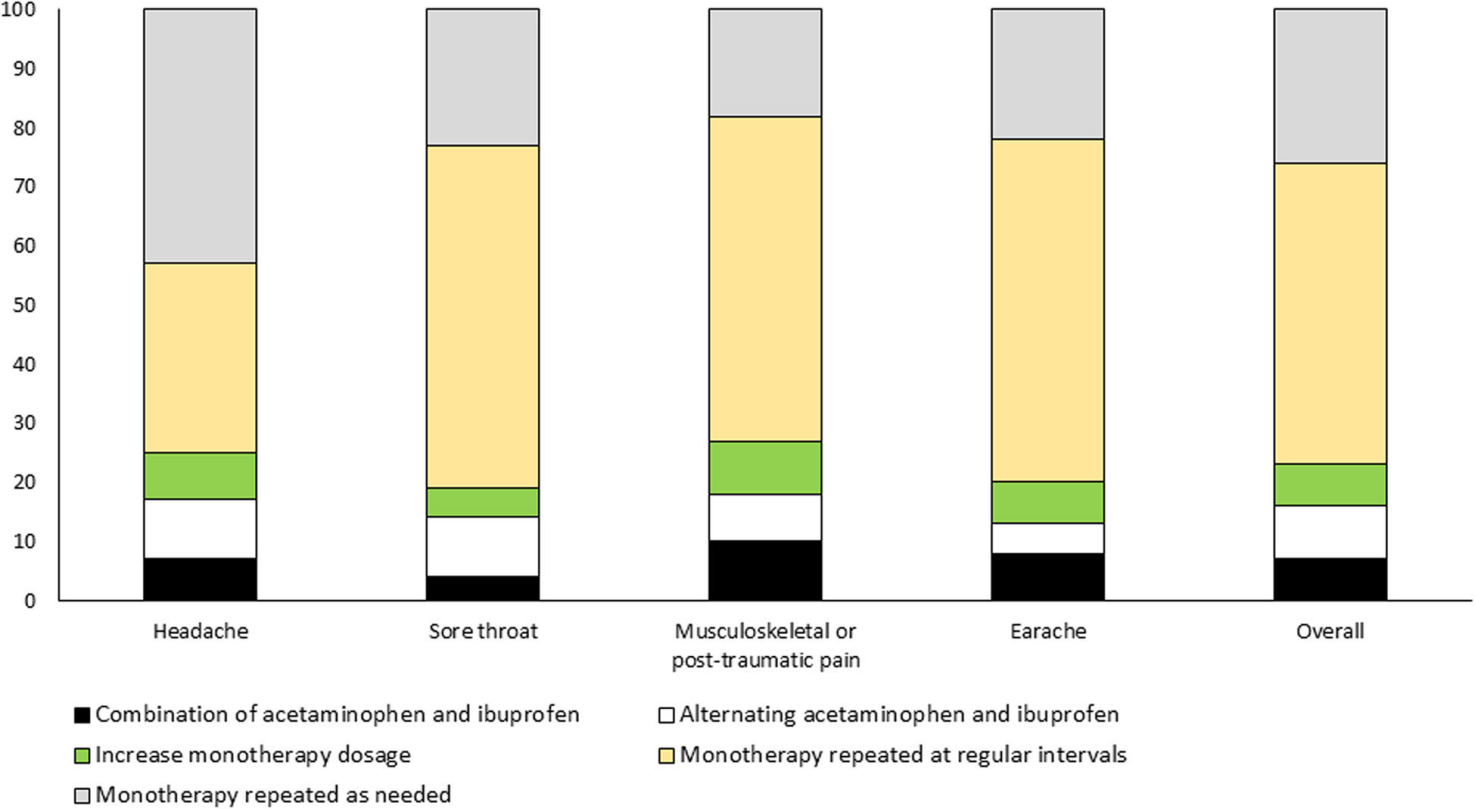
Therapeutic regimen of non-opioid analgesics prescribed to treat persistent mild-moderate pain

Finally, in case of persistent mild-moderate pain (i.e. only for pediatricians who had chosen the answers 1a, 2c or 2d), the therapeutic scheme most frequently adopted for non-opioid analgesics (*question* 1*6*) was monotherapy repeated at regular intervals in 763/1512 (50.5%) cases or as needed in 397/1512 (26.3%; *p* < 0.001 for both) (Fig. 4). Notably, in 108 (7.1%) cases, ibuprofen was used in combination with acetaminophen, and in 136 (9.0%) the two drugs were alternated. Similar results were observed for all types of pain, except for headache, for which monotherapy repeated as needed was employed in 42% of cases, followed by monotherapy repeated at regular intervals in 32% of cases.

#### Moderate-severe pain

**Fig. 5:**
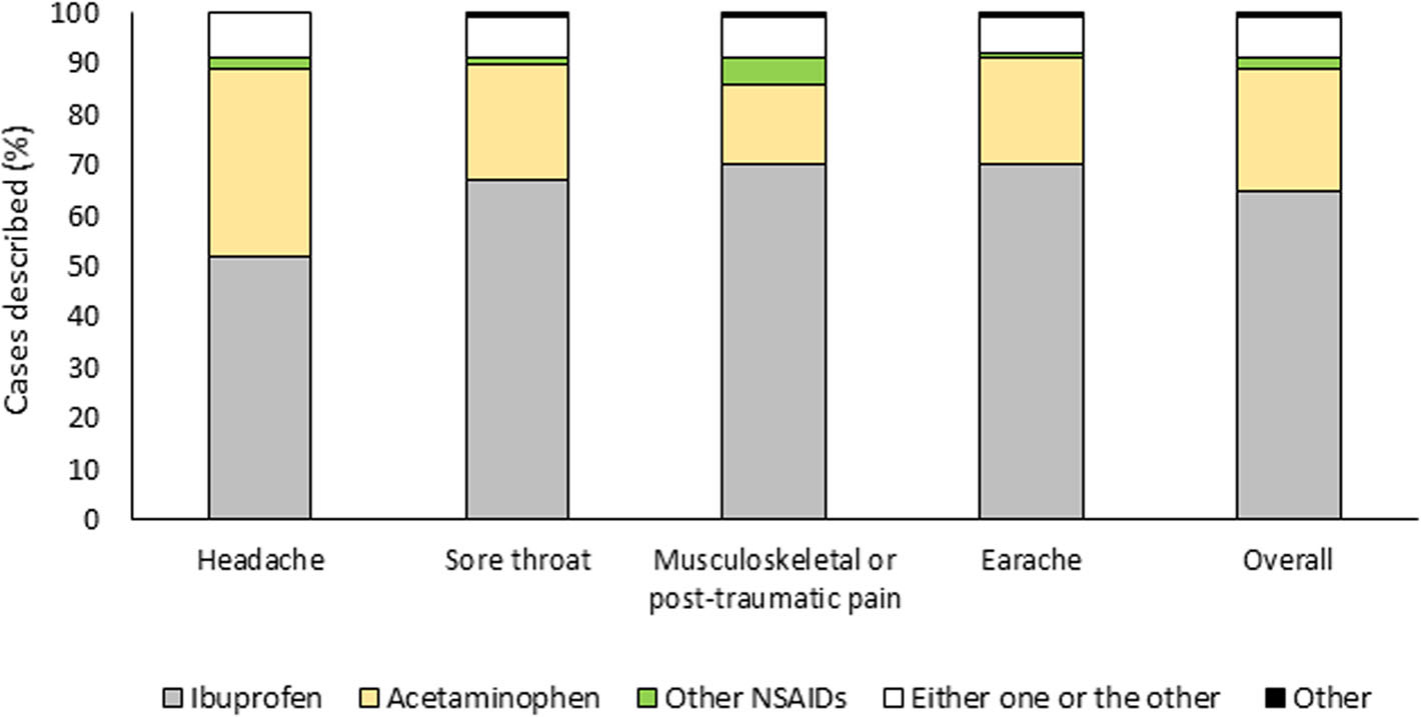
Analgesics used in combination with weak opioids to treat moderate-severe pain not completely controlled by monotherapy

In case of moderate to severe pain not completely controlled by opioid monotherapy, the agents most frequently prescribed in combination with weak opioids (*question 17*) were ibuprofen in 2980/4570 (65.2%) cases and acetaminophen in 1084/4570 (23.7%); the data considered as a whole and by pain type are reported in Fig. 5. Ibuprofen was the most frequent choice for all pain types, whereas acetaminophen was more frequently prescribed for headache, although less than ibuprofen (37.0% vs 51.2%, respectively) (*P* < 0.001).

## Discussion

Pediatric pain is an important public health problem, even in apparently healthy subjects, due to both its frequency and impact on daily living [19, 21]. The risk to underestimate these aspects is high, owing in part to the scarce use of appropriate assessment tools. The present study provides information on the attitude and practice of Italian family pediatricians in the management of acute pain in children aged 7 to 12 years. The types of acute pain addressed in our analysis were equally distributed across headache, sore throat, musculoskeletal/post-traumatic pain and earache, and were mostly of mild-moderate intensity and short-lasting. Altogether, the survey highlighted a poor adherence to current recommendations with regard to the use of algometric scales for measuring pain and to the treatment approach.

National and International guidelines mandate the measurement and relieve of pain in all children and in all instances, as painful stimuli not adequately evaluated and treated in the pediatric age may have important effects on the short- and long-term health outcome of young patients [4, 6–8]. Optimal care requires assessment of pain intensity through a psychometrically validated scale, along with the use of the most appropriate medication and dosage for each individual patient. In the present study, however, only half of participants declared to employ an algometric scale to measure pain, which were mainly used for headache and musculoskeletal pain, highlighting the urgent need to educate physicians on the importance of using these tools. This observation is alarming if one considers that the survey was conducted more than 7 years after the Italian law 38/2010 had entered into force [22], and that several promotional initiatives were carried out since then. Among these, a survey targeting 4900 Italian family pediatricians had shown that adequate training may help increase the awareness on optimal management of pain [20]: however, despite these efforts, the percentage of pediatricians using an algometric scale in the present study remained unchanged.

Regarding the therapeutic approach, 60.5% of the participants stated that they always prescribe an analgesic therapy because pain constitutes a negative experience for the child. This finding, together with the observation that quality of life impairment and pain intensity were the main reasons for deciding to start a therapy, suggests a high sensitivity of the pediatrician towards the negative physical as well as emotional impact of pain, in line with the principles of the Italian law 38/2010 [22]. However, nearly 1 in 3 pediatricians prescribed an analgesic drug only for some types of pain, generally to avoid that the child experiences intense pain, rather than for each instance of pain.

National and International guidelines recommend both ibuprofen or acetaminophen as first-line treatment for acute pain with the same level of evidence, as they have comparable efficacy and safety profile [4, 7, 23–25]. However, the similar efficacy of acetaminophen and ibuprofen in musculoskeletal trauma, orthopedic pain and headache is still insufficiently documented. Available evidence supports ibuprofen as an adequate first-line option in the case of musculoskeletal trauma [26] and a better analgesic compared to acetaminophen in this type of pain [27] and headache as well (evidence from small studies [28, 29]). In the management of sore throat and earache, both ibuprofen and acetaminophen showed an equivalent control of pain, even if ibuprofen could be preferred because of its inflammatory properties [30]. In the present study, ibuprofen and acetaminophen were the most commonly used first-line non-opioid drugs in case of mild-moderate pain, with a slight preference for the former (53.3% vs 44.4%, respectively), in line with the above studies [30–32].

One interesting finding regards the dosage prescribed for both ibuprofen and acetaminophen: indeed, in all children treated with ibuprofen and in 94.7% of those treated with acetaminophen, the dosage was in line with current recommendations, whereas in 5.3% of cases acetaminophen was overdosed [4]. Considering that in a previous survey conducted among family pediatricians in Italy, only 13.5% of pediatricians had prescribed acetaminophen at the correct dosage [20], our results are reassuring; still, they pinpoint the need for further efforts to fully optimize the management of pain in children also in light of the fact that, in case of overdose, the toxicity of acetaminophen occurs quicker and is more difficult to manage as compared with ibuprofen overdose [33]. This is likely due to the wider therapeutic window of ibuprofen compared to that of acetaminophen [33, 34].

In case of mild-moderate pain, efficacy was the most relevant aspect considered in the choice of ibuprofen, which was preferred over acetaminophen or any other analgesic also for its anti-inflammatory activity. In contrast, better tolerability was reported as more relevant when prescribing acetaminophen, even though current evidence and guidelines indicate that the safety profile of the two drugs is similar, as no substantial differences in the incidence of adverse events have been reported [30, 33].

Indeed, ibuprofen has a good safety profile in terms of both gastric and renal tolerability similar to the profile of acetaminophen [23, 24, 30, 33]. In one of the largest trials of ibuprofen and acetaminophen, the risk of gastrointestinal bleeding was low (7.2 per 100,000), with no statistically significant difference between the two treatment groups (*p* = 0.31) [30]. Other studies have confirmed that upper gastrointestinal complications (UGIC’s) are rare events in children treated with NSAIDs, with a low absolute risk of about 2.4 UGIC incidents per 10,000 children at emergency departments ([35]). Bianciotto and coworkers reported an adjusted OR for the risk of UGICs in NSAID-treated children of 8.2 (95% CI 2.6–26.0), with one-third of cases attributable to exposure to NSAIDs administered at therapeutic doses [35]. However, no significant difference was found in the risk of UGICs between acetaminophen and ibuprofen (adjusted OR 2.0, 95% CI 1.5–2.6 and 3.7, 95% CI 2.3–5.9, respectively), although UGICs were lower in children treated with acetaminophen [35]. In the present study, as much as 52.5% of responders stated that they had recommended to take ibuprofen after meals always because of the fear of gastric side effects. However, it must be kept in mind that the administration with food slows the speed of absorption and may, thus, reduce efficacy, possibly leading to more frequent administrations and to the risk of over-dosing. For this reason, some authors advise to take over-the-counter ibuprofen on a fasting stomach to achieve a rapid onset of action and effect, thereby avoiding the use of an ‘extra’ dose [33, 36–38]. The practice to take it after meals should be limited to selected at-risk cases or in case of prolonged administration.

As for the renal function, it has been shown that, in children with normal function and effective circulating volume, ibuprofen and acetaminophen are equally safe and renal conditions are unlikely to occur [30, 33]. Caution is recommended in children with high dehydration levels and pre-existing renal disease, since in these patients NSAIDs can be associated to an increased risk of acute kidney injury even at the therapeutic doses [39, 40], as a result of NSAID-mediated inhibition of renal prostaglandins [41]. In any case, keeping the patient hydrated may help preventing dehydration and any kidney toxicity.

On the other end, the concomitant administration of the two molecules may increase the risk of renal toxicity due to their synergistic effect on renal function [30] (adjusted reporting odds ratios: 4.01 [95%CI: 2.96–5.43] [42]). For this reason, the practice to combine or alternate acetaminophen and ibuprofen must be discouraged and avoided whenever not strictly necessary. On the other hand, due to the ban on codeine and the FDA black warning on the use of tramadol under 12 years of age, the options of a family physician for the treatment of a moderate-to-severe pain resistant to first-line therapy are very limited, considering that the administration of a major opioid in this setting would be unlikely. Some evidence from adult and pediatric literature suggests that the association between acetaminophen and ibuprofen is both efficacious and safe. Indeed, although pediatricians should be fully aware of the potential risk factors related to the concomitant use of acetaminophen and ibuprofen, especially in case of dehydration, malnutrition, coexistent liver or gastric disease or administration of other drugs, the association of these two medications maintains a reasonable cost-benefit ratio in many situations of refractory pain. In this context, evidence exists supporting the feasibility of the association of the two drugs in some situations [43–45].

In the present survey, in case of mild-moderate pain persisting for a few days or a week, monotherapy (either repeated at regular intervals [50.5%] or as needed [26.3%]) was the most frequently prescribed therapeutic regimen. However, in 16.1% of cases ibuprofen and acetaminophen were used in combination or alternated. Despite the association could maintain a reasonable cost-benefit ratio in some specific cases of non-responding pain or fever, pediatricians must be fully aware of the possible risk factors coming from the combination of pathophysiological aspects of the two drugs and that selecting the most suitable active substance for the individual child may help optimizing the chance of treatment success already upfront, thereby limiting the need to administer further treatments. In this context, parents and caregivers should receive clear indications about the prescribed regimen.

Regarding the management of moderate-severe pain not completely controlled by opioids, ibuprofen and acetaminophen were the most used add-on medications, with ibuprofen being much more prescribed than acetaminophen (65.2% vs 23.7%, respectively), overall and in all pain types. This result is interesting, as ibuprofen and acetaminophen have been shown to have less adverse events than opioids in the treatment of acute pain in children [25, 30] and, therefore, adding non-opioids to opioid medications may help reducing the use and, hence, the toxicity of the latter. For this reason, in case of moderate-severe pain, opioid medications should be added to, rather than replace, non-opioids [25]. However, non-opioids or opioids should always be used by taking into account their benefit/risk ratio profile. In this context, specific cases must be considered, in whom the cause of pain may depend on other rare conditions, such as headache related to intracranial pressure or meningoencephalitis. Furthermore, it must be considered that even if non-opioids as ibuprofen and acetaminophen are recommended as first-line treatment, in some instances of sore throat or headache caused by bacterial infections, antibiotic therapy must be used following appropriate diagnosis.

Finally, one finding that deserves attention regards some pharmacological characteristics not considered as relevant by the responders when choosing a painkiller: manageability (i.e. wide therapeutic window) [33] and therapy compliance [46]. Compliance is a key element to ensure the maximum efficacy of prescribed medications. Among the factors that may affect therapy compliance in pediatrics, the following are particularly important: the pharmaceutical form, which varies according to the child’s age, clinical characteristics and needs; drug palatability, particularly important in the case of oral formulations (which are the most appropriate for the children described in the present study); dose volume and number of administrations per day, which should be limited to make therapy more acceptable [46].

The current study has some limitations, which include the cross-sectional design, the lack of validated questions of the survey and a possible recall bias. We acknowledge that pain was only measured by a conventional pain scale. Owing to the multifactorial nature of this symptom, a thorough assessment would require the evaluation of a broad range of factors, which include physical, social and school activities, psychological aspects, sociocultural contexts, family and peer interactions, cognitive functioning, emotional distress, mood, behavior, and pain-coping strategies [47]. Still, the tool for pain measurement used in the study was selected and agreed upon by all participating investigators. We know and understand that any instrument used to measure pain can be criticized. However, because to used tool is well validated and widely used in the literature, we are confident that the results of pain assessment in our study are reliable and valid. The main strengths of our analysis are the high number of participants and their homogeneous distribution across Italy, which make the results of the survey on pain management generalizable to the Italian population of family pediatricians.

## Conclusions

In the management of acute pain, current recommendations advocate the constant measurement of intensity through algometric scales and the administration of ibuprofen and acetaminophen as first line treatment. In this context, the careful tailoring of the therapeutic strategy is crucial to guarantee treatment compliance, ultimately optimizing pain relief. The present survey revealed several gaps between current practice and recommendations among Italian family pediatricians, with regard to both assessment and therapeutic approaches of different types of acute pain. Our results emphasize the need to raise awareness on the possible exposure of the child to an increased risk of adverse events and of short- and long-term consequences in case of suboptimal pain management. Although in the last decade several improvements have been achieved in Italy, further efforts are required to provide children with the most adequate care.

## Supplementary information

**Additional file 1.** The 17-question survey translated in English.

## Data Availability

All data generated or analyzed during this study are included in this published article [and its supplementary information files]. The datasets used and/or analyzed during the current study are available from the corresponding author on reasonable request.

## Abbreviations

CME: continuing medical education
IQR: interquartile range.
SD: standard deviation
