# Supplementary material for: Acute pain management in children: a survey of Italian pediatricians
Source: Ital J Pediatr. 2019 Dec 3;45:156. doi: 10.1186/s13052-019-0754-3 (PMC6892238; doi:10.1186/s13052-019-0754-3)
Supplement: Supplementary file 1 — Additional file 1. The 17-question survey translated in English. [file 13052_2019_754_MOESM1_ESM.docx]

**Survey**

Dear Doctor,

Welcome to the pediatric pain management survey.

We remind you that the age group taken into consideration for the case studies is between 7 and 12 years, and that for the participation in the FAD course it is necessary to fill in at least 2 questionnaires for each type of pain in the survey below ( headache, sore throat, musculoskeletal or post-traumatic pain, and earache).

**Choose the kind of pain of the case study you are filling in the questionnaire for**:

- headache
- sore throat
- musculoskeletal or post-traumatic pain
- earache

_______________________________________________________________________________________

**Identification of the intensity and duration of the pain considered:**

1) The pain intensity is:

a) Mild

b) Moderate

c) Severe

2) The pain lasts from:

a) A few hours

b) 1 day

c) Some days

d) At least 1 week

_______________________________________________________________________________________

**General information on the case study into consideration:**

3) Age ................ (age range from 7 to 12 years)

4) Sex

- M
- F

_______________________________________________________________________________________

**Evaluation of pain**

5) Did you use an algometric scale for the clinical evaluation of pain in this case?

- Yes
- No

6) What are your thoughts on the choice regarding the algometric scale?

a) Scales should be used in every patient with pain symptoms

b) Scales should be used only for preverbal children

c) Scales should be used only in the child capable of self-assessment

d) Scales should be used according to the referred pain intensity

e) Scales should be used rarely

_______________________________________________________________________________________

**Therapeutic approach**

7) In general, on what principles do you base your choice of treating pain in children?

a) I prescribe a pain therapy only for some types of pain to prevent the child from experiencing intense painful sensations

b) I always prescribe therapy for pain because the experience of pain is always negative for the child

c) I never prescribe any treatment for mild pain

d) I do not prescribe therapy for mild to moderate pain because I think the side effects of the therapy are more than its benefits

e) I never prescribe any early pain therapy because it can mask the diagnosis

8) On which criteria have you based your decision to undertake an analgesic pharmacological therapy for this case? Choose a value from 1 to 5, where 1 is not important and 5 very important.

|  | 1 | 2 | 3 | 4 | 5 |
| --- | --- | --- | --- | --- | --- |
| a) Duration of pain |  |  |  |  |  |
| b) Origin of pain |  |  |  |  |  |
| c) Impact of pain on quality of life |  |  |  |  |  |
| d) Pain intensity |  |  |  |  |  |

9) What aspects have you considered when choosing between non-opioid first-line drugs for mild-moderate pain? Choose a value from 1 to 5, where 1 is not important and 5 very important.

(only for answers 1a and 1b- mild and moderate pain)

| a) Age of the child | 1 | 2 | 3 |  | 4 | 5 |
| --- | --- | --- | --- | --- | --- | --- |
| b) Possible comorbidity |  |  |  |  |  |  |
| c) General condition of the child |  |  |  |  |  |  |
| d) Possible concomitant therapies |  |  |  |  |  |  |

10) For the present case which active ingredient has been used as an oral non-opioid drug for analgesic purposes?

(only for answers 1a and 1b- mild and moderate pain)

a) Acetaminophen

b) Ibuprofen

c) Ketoprofen lysine salt

d) Naproxen

e) Other (specify ...............)

11) What dosage? (posology presented only for the drug selected in question 10)

Acetaminophen

a) 10 mg / kg every 4-6h

b) 15 mg / kg every 4-6h

c) ≥15 mg / kg every 4-6h

Ibuprofen

a) 5 mg / kg every 6-8 h

b) 10 mg / kg every 6-8 h

Ketoprofen lysine salt

a) 40 mg 1 time a day

b) 40 mg 2 times a day

c) 40 mg 3 times a day

Naproxen

a) 220 mg 1 time a day

b) 500 mg 1 time a day

Other

(specify dosage ...............)

12) In children with mild to moderate pain, which are the reasons that oriented the prescription towards______? (drug name filled in based on the drug selected in question 10)

Choose a value from 1 to 5, where 1 is not important and 5 very important.

Acetaminophen?

| a) Established therapeutic practice | 1 | 2 | 3 | 4 | 5 |
| --- | --- | --- | --- | --- | --- |
| b) Parental satisfaction / experience |  |  |  |  |  |
| c) Effectiveness |  |  |  |  |  |
| d) Better tolerability |  |  |  |  |  |
| e) History of asthma |  |  |  |  |  |
| f) Recommendations contained in the guidelines |  |  |  |  |  |

Ibuprofen?

| a) Established therapeutic practice | 1 | 2 | 3 | 4 | 5 |
| --- | --- | --- | --- | --- | --- |
| b) Parental satisfaction / experience |  |  |  |  |  |
| c) Effectiveness |  |  |  |  |  |
| d) Better tolerability |  |  |  |  |  |
| e) History of asthma |  |  |  |  |  |
| f) Recommendations contained in the guidelines |  |  |  |  |  |

ketoprofen lysine salt?

| a) Established therapeutic practice | 1 | 2 | 3 | 4 | 5 |
| --- | --- | --- | --- | --- | --- |
| b) Parental satisfaction / experience |  |  |  |  |  |
| c) Effectiveness |  |  |  |  |  |
| d) Better tolerability |  |  |  |  |  |
| e) History of asthma |  |  |  |  |  |
| f) Recommendations contained in the guidelines |  |  |  |  |  |

naproxen?

| a) Established therapeutic practice | 1 | 2 | 3 | 4 | 5 |
| --- | --- | --- | --- | --- | --- |
| b) Parental satisfaction / experience |  |  |  |  |  |
| c) Effectiveness |  |  |  |  |  |
| d) Better tolerability |  |  |  |  |  |
| e) History of asthma |  |  |  |  |  |
| f) Recommendations contained in the guidelines |  |  |  |  |  |

other?

| a) Established therapeutic practice | 1 | 2 | 3 | 4 | 5 |
| --- | --- | --- | --- | --- | --- |
| b) Parental satisfaction / experience |  |  |  |  |  |
| c) Effectiveness |  |  |  |  |  |
| d) Better tolerability |  |  |  |  |  |
| e) History of asthma |  |  |  |  |  |
| f) Recommendations contained in the guidelines |  |  |  |  |  |

13) For which reasons have you prescribed ibuprofen compared to acetaminophen or another painkiller?

(only for those who answered 10b- ibuprofen)

a) None, in particular I consider them substantially equivalent

b) Inflammation/infectious origin of pain

c) Lower risk of overdose

d) Less hepatotoxicity

e) Greater analgesic efficacy

f) Availability of different pharmaceutical formulations and dosages

g) A lower frequency of administration

h) Recommendations contained in the guidelines

14) In the case your prescription has been ibuprofen orally, did you recommend taking it on a full stomach? (only for those who answered 10b- ibuprofen)

a) No, I do not consider it necessary for occasional use (when needed)

b) Yes, always, to prevent gastritis

c) Yes, for persistent pain that requires multiple drug administrations

d) Yes, but only in case of long-term therapy

e) Yes, but only in children with a history of gastrointestinal disorders

15) For the prescription of a non-opioid analgesic, which pharmacological characteristics have weighed more on the choice of the molecule for the clinical case in question? (only for answers 1a and 1b- mild and moderate pain)

a) Analgesic efficacy

b) The rapidity of the analgesic effect

c) Duration of analgesic action

d) Tolerability profile

e) Manageability (width of the therapeutic window)

f) Ease of dosage and safety

g) palatability

16) For persistent mild-moderate pain in the case in question, which therapeutic scheme have you prescribed for non-opioid analgesics?

(only for answers 1a 2c or 2d - mild pain that lasts a few days or a week)

a) Combination therapy with acetaminophen and ibuprofen

b) Alternating acetaminophen and ibuprofen

c) Increase the dosage of monotherapy

d) Monotherapy repeated at regular intervals

e) Monotherapy repeated as needed

17) In case of moderate-severe pain not completely controlled by monotherapy for the child in question, which molecule have you considered most useful to associate with weak opioids?

(only for answers 1b and 1c - moderate, severe pain)

a) Acetaminophen

b) Ibuprofen

c) Either one or the other

d) Other NSAIDs

e) Other (specify ...............)
